# Supplementary figures and images for: SIN-3 coregulator maintains adaptive capacity to different diets in Caenorhabditis elegans through vitamin B12
Source: G3 (Bethesda). 2026 Apr 11;16(6):jkag087. doi: 10.1093/g3journal/jkag087 (PMC13232490; doi:10.1093/g3journal/jkag087)

Figure S3

a

| gene          | Log2FC | pvalue                |
|---------------|--------|-----------------------|
| <i>hach-1</i> | -0.185 | $6.47 \cdot 10^{-4}$  |
| <i>hphd-1</i> | -0.763 | $2.76 \cdot 10^{-5}$  |
| <i>alh-8</i>  | -0.887 | $3.25 \cdot 10^{-29}$ |

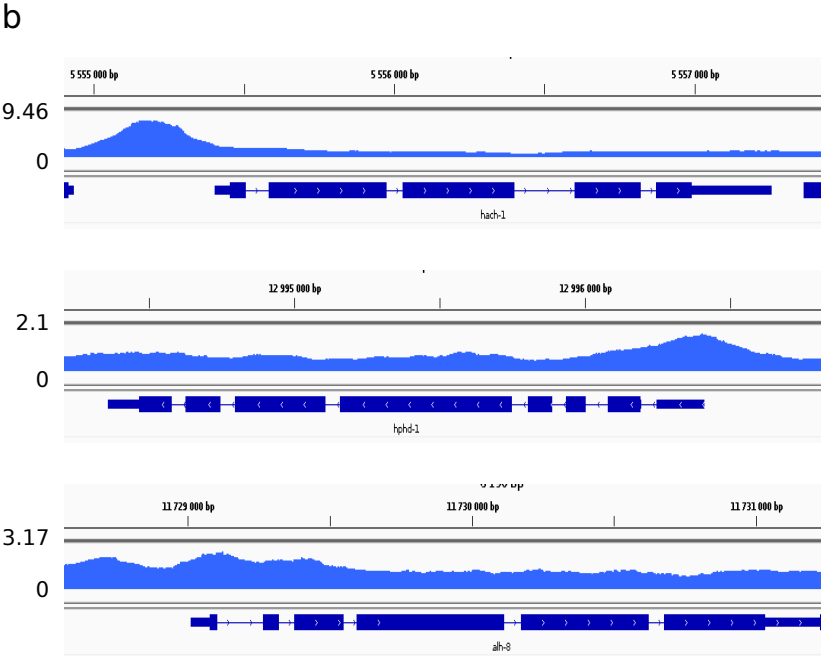

Supplement: jkag087_Supplementary_Data [file jkag087_supplementary_data.zip › figure_S3_final.pdf]

Figure S4

a

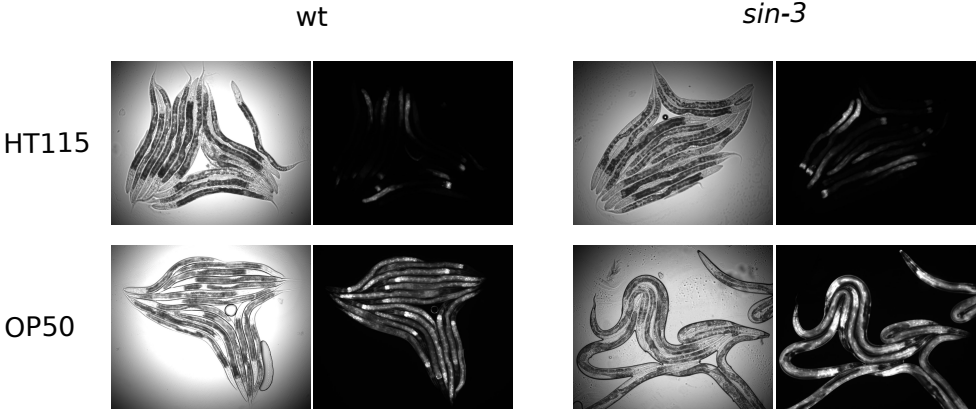

b

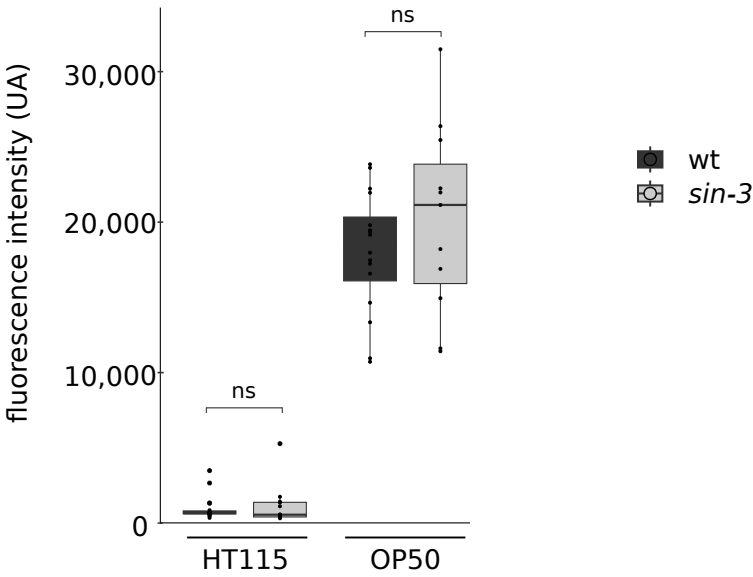

Supplement: jkag087_Supplementary_Data [file jkag087_supplementary_data.zip › figure_S4_final.pdf]

Figure S5

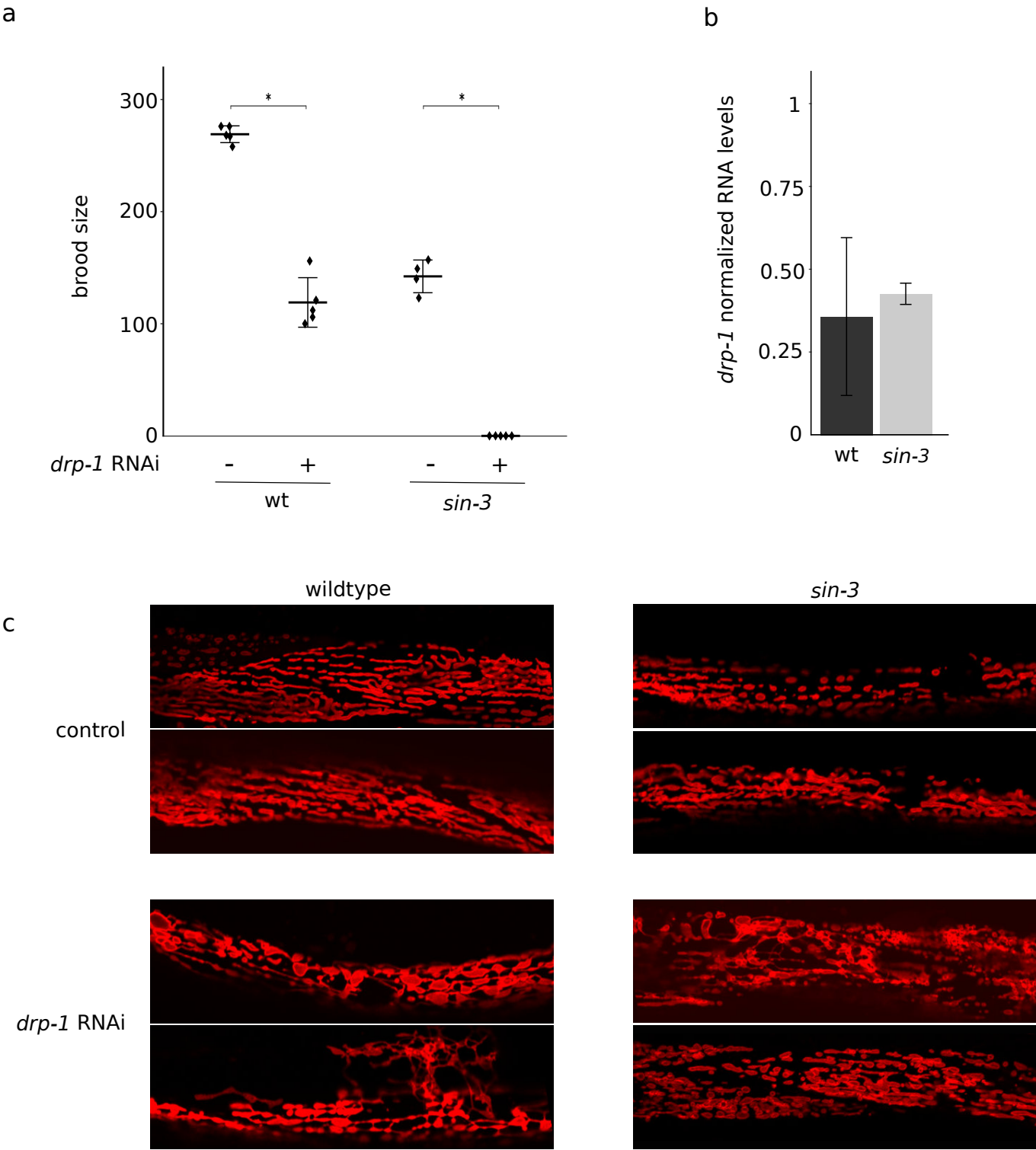

Supplement: jkag087_Supplementary_Data [file jkag087_supplementary_data.zip › figure_S5_final.pdf]

Figure S1

a

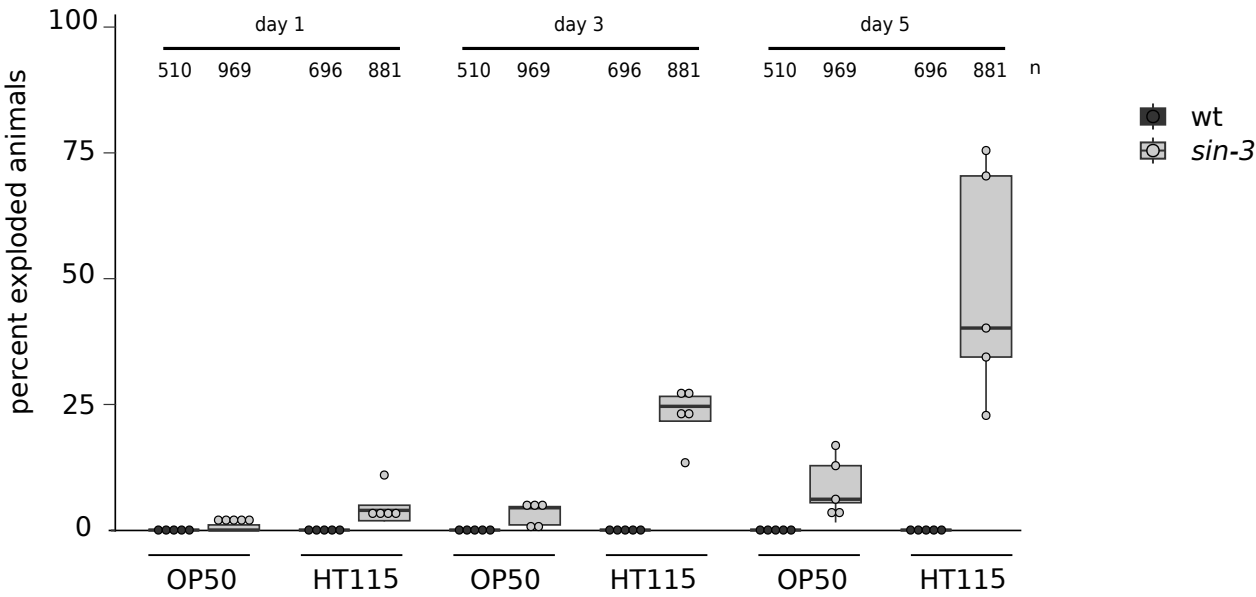

b

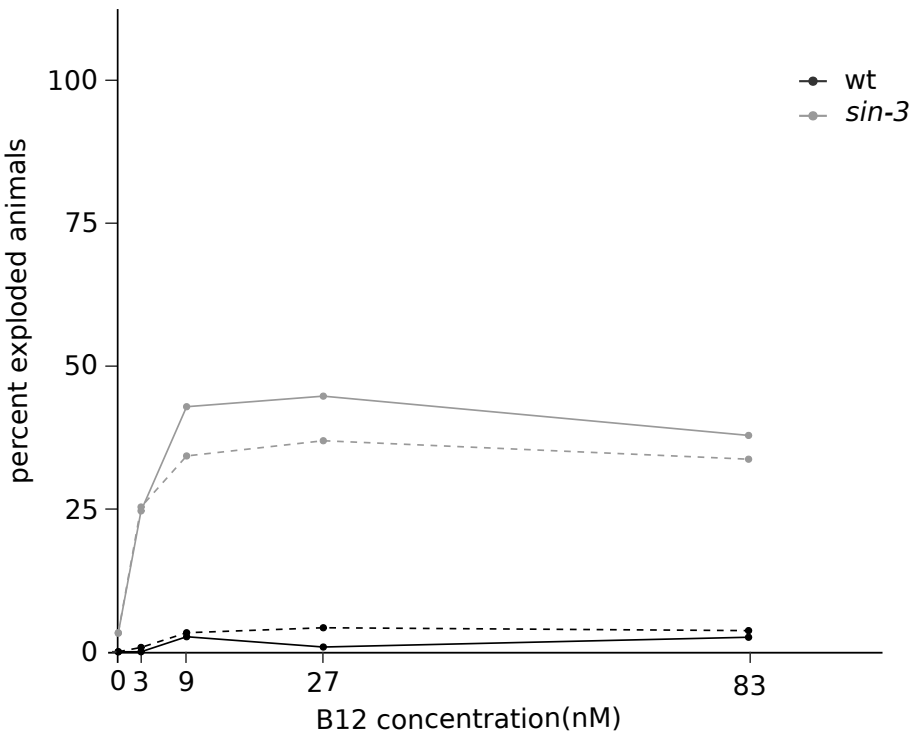

Supplement: jkag087_Supplementary_Data [file jkag087_supplementary_data.zip › figure_S1_final.pdf]

Figure S2

a *metr-1* RNAi

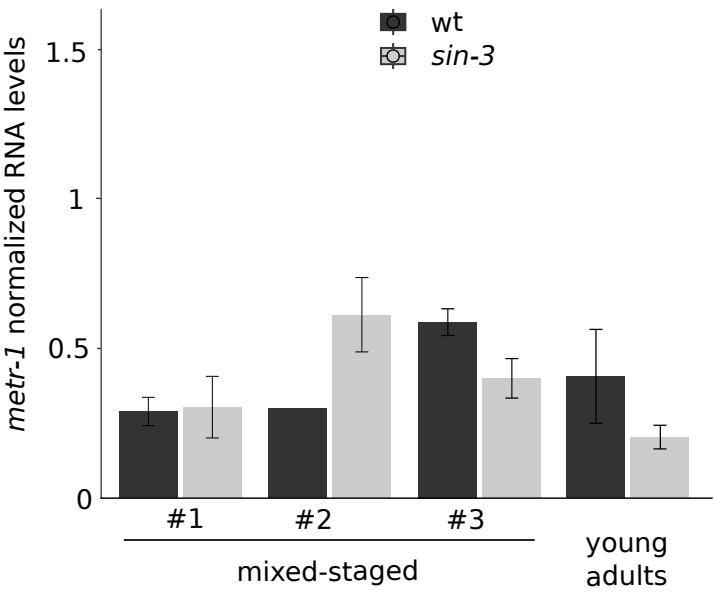

b *mmcm-1* RNAi

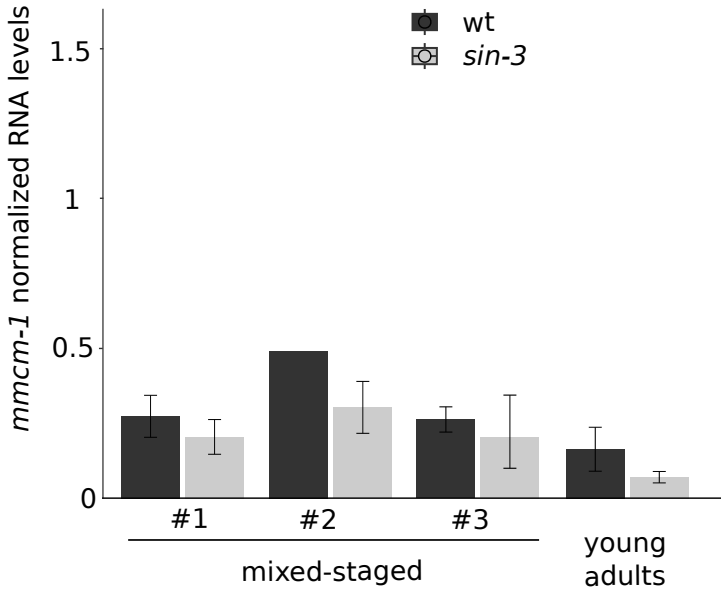

Supplement: jkag087_Supplementary_Data [file jkag087_supplementary_data.zip › figure_S2_final..pdf]
