# Supplementary material for: SIN-3 coregulator maintains adaptive capacity to different diets in Caenorhabditis elegans through vitamin B12
Source: G3 (Bethesda). 2026 Apr 11;16(6):jkag087. doi: 10.1093/g3journal/jkag087 (PMC13232490; doi:10.1093/g3journal/jkag087)
Supplement: Supplementary file 5 [file jkag087_supplementary_data.zip › Supplemental_Figure_Legends_G3-2025-406337.odt]

Supplementary Figures legends

Figure S1. Both ageing and B12 supplementation on OP50 have an effect on survival of sin-3 mutant animals (A) Proportion of sin-3 mutant adult animals grown at 20°C either on OP50 or HT115 bacterial strains presenting an exploded phenotype, at day1  (left) , day 3 (middle) or day 5 (right) of adulthood. N = total number of worms scored. (B) Dose-response curve showing exploded phenotype on increasing B12 concentration on OP50. Two independent experiments were performed as presented on the graph. For each concentration a total of 200 – 300 worms per strain were scored. 

Figure S2. RT-qPCR analysis of metr-1 (A) and mmcm-1 (B) gene levels after RNAi. RNA was extracted from sin-3 or wildtype mixed stages animals grown on RNAi plates for at least two generations. RNA levels were normalized to the mean of act-1 and cdc-42 gene levels (except for experiment # 2 for metr-1 where normalization was done with cdc-42 only). Two technical replicates were performed for each of three independent RNAi experiments.

Figure S3. Effect of drp-1 inactivation by RNAi on mitochondria morphology and brood size of wildtype and sin-3 animals. (A) Brood size count of wild type (wt) and sin-3 mutants animals after RNAi treatment against drp-1. Pairwise comparisons were carried out using Kruskal-Wallis non parametric statistical test. * P<0.05. (B) RT-qPCR analysis of drp-1 gene levels after RNAi for one generation. Two technical replicates were performed and normalization was done using the mean of cdc-42 and act-1 genes. (C) Representative confocal images of mitochondria morphology in body wall muscles expressing myo-3p::tomm-20Nter::wrmScarlet in wildtype and sin-3 mutant animals at day 4 of adulthood. 

Figure S4. acdh-1 induction occurs normally on OP50 in sin-3 mutant animals. (A). Fluorescence and bright field representative images of wildtype and sin-3 young adult animals expressing acdh-1p::GFP grown either on OP50 or HT115. Images were taken with  the same exposition time for all conditions.  (B) Quantification of GFP fluorescence intensity from animals presented in (A).

Figure S5. SIN-3 directly regulates the expression of genes involved in the shunt propionate pathway. (A) hach-1, hphd-1 and alh-8 gene levels coming from transcriptomics analysis of sin-3 mutant germlines (Giovannetti et al, elife 2024, GSE227498). (B) IGV snapshots showing z-scored BEADS normalized SIN-3 ChIP-seq signals from wildtype embryos on hach-1, hphd-1 and alh-8 genes (Beurton et al, NAR 2019, GSE114715).
